# Central Placenta Previa With Coexisting Central Cervical Fibroid in Pregnancy: An Obstetrician’s Nightmare

**DOI:** 10.7759/cureus.15910

**Published:** 2021-06-24

**Authors:** Archana Barik, Vinita Singh, Anisha Choudhary, Preeti Yadav

**Affiliations:** 1 Obstetrics and Gynecology, Tata Main Hospital, Jamshedpur, IND

**Keywords:** placenta previa, postpartum, hemorrhage, cervical, fibroid, pregnancy, antepartum

## Abstract

Pregnancy with central placenta praevia and a coexistent cervical fibroid is infrequently encountered in clinical practice. A patient with this unusual combination is a nightmare for any clinician, especially if she presents with obstetric emergencies. In this scenario, there is a high chance of catastrophic obstetric hemorrhage during the peripartum period leading to a fatal outcome. We report a case of a 27-year-old lady at term pregnancy, who was brought to emergency in labor pain with antepartum hemorrhage. Subsequently, she was diagnosed to have central placenta praevia along with a large central cervical fibroid. An emergency cesarean section was performed to deliver the child. Intraoperatively, she had a major postpartum hemorrhage which was successfully managed with intrauterine balloon tamponade, hemostatic sutures, and uterine artery ligation. We could avoid cesarean hysterectomy by precise anticipation, meticulous planning, and step-wise protocol-driven management.

## Introduction

One of the most important causes of maternal morbidity and mortality all over the world is obstetric hemorrhage. Placenta praevia is known to engender life-threatening bleeding in the third trimester and contributes to one-fifth of cases of antepartum hemorrhage [1]. It is characterized by abnormal implantation of the placenta in the lower uterine segment, fully or partially covering the internal os. The overall prevalence of placenta previa is 5.2 per 1,000 pregnancies. In Asian countries, the number goes up to 12.2 per 1,000 pregnancies [2].

Uterine fibroid has been speculated to be one of the risk factors of abnormal placental implantation. Studies have found that the risk of placenta previa increases two to three-fold in cases of associated fibroid uterus [3,4]. Cervical fibroid an uncommon variant of uterine fibroid may infrequently associate with pregnancy [5]. On occasions, they can significantly affect the course and outcome of pregnancy. Infection, urinary retention, preterm labor, obstructed labor, and increased incidence of hemorrhage are some of the known complications [6]. It is still unknown whether cervical fibroid has got any role in the development of placenta previa. Nevertheless, cervical fibroid in pregnancy complicated by placenta previa is a challenging scenario for obstetricians.

We encountered a unique case where a large central cervical fibroid was associated with central placenta previa, who presented to us in active labor with antepartum hemorrhage.

## Case presentation

A 27-year-old woman at 37 weeks and two days of gestation got admitted to the labor room with pain abdomen and bleeding per vaginum. She was a second gravida with no living issue. Before one and a half years back, she had a preterm twin vaginal delivery at 36 weeks of gestation with early neonatal death of both babies.

In the current pregnancy, a prior obstetric ultrasound done at 20 weeks showed a myoma measuring 11.5 cm x 8.5 cm in the cervical region and the placenta occupying the right lateral position of the uterus. The patient was not under regular antenatal check-ups. She had no other associated comorbidities.

**Figure 1:**
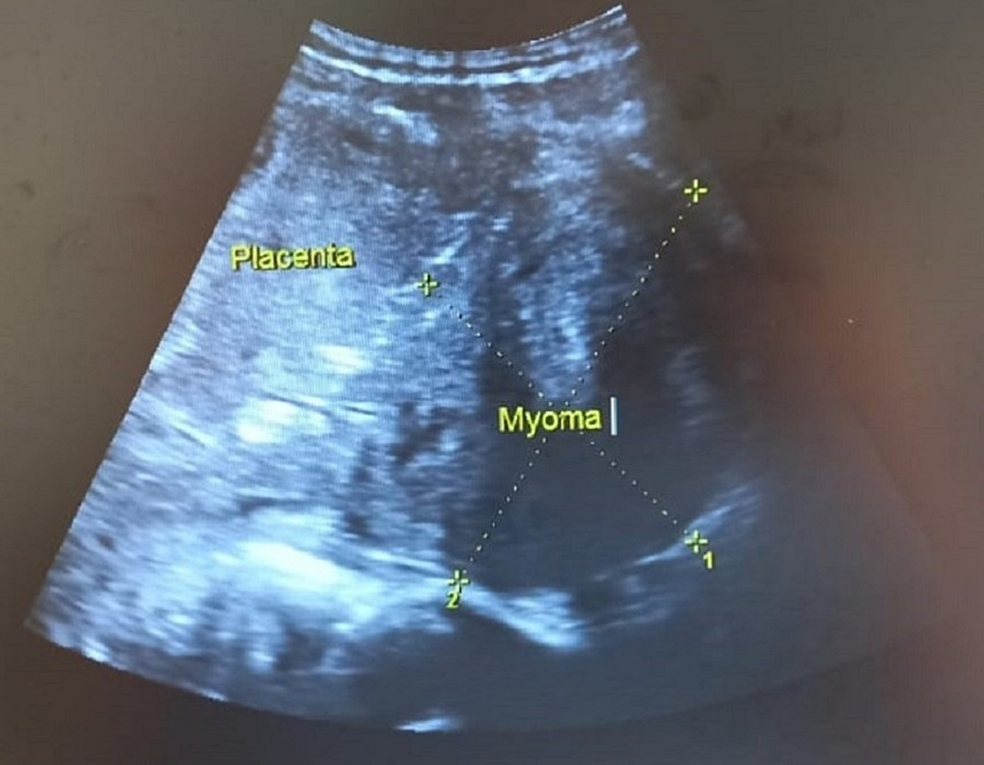
Abdominal ultrasound image showing central placenta previa and a cervical fibroid or myoma of size 10 cm x 8 cm

**Figure 2:**
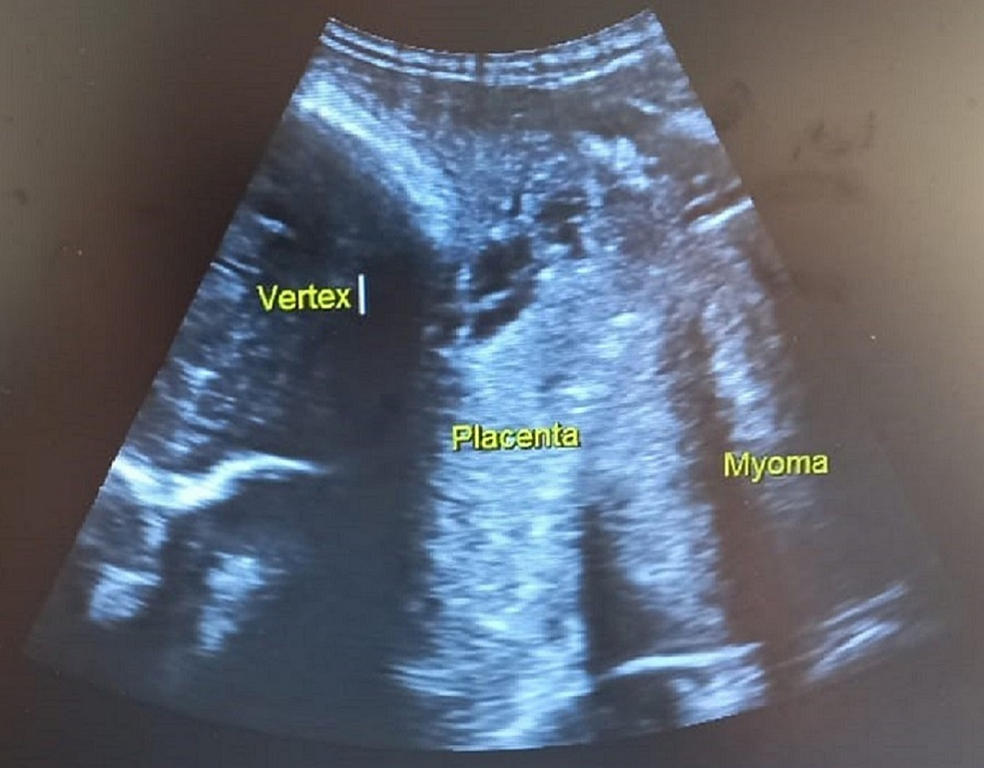
Abdominal ultrasound image showing the head of the fetus, central placenta previa, and the cervical fibroid or myoma

On general examination, she was pale, but her vitals were stable. Obstetrical examination revealed a uterus of term size with mild contractions, and there was moderate bleeding per vaginum. An ultrasound examination was done, which showed a central placenta previa and a central cervical fibroid of size measuring 10 cm x 8 cm (Figures 1, 2). The fetus was in cephalic presentation with an estimated weight of 3.1 kg. The amniotic fluid was of adequate amount. The blood investigations showed anemia with hemoglobin of 7.8 gm%, and liver enzymes were deranged suggestive of obstetric cholestasis. The rest of the blood investigations were within normal limits.

She was planned for an emergency cesarean section because of central placenta praevia with antepartum hemorrhage. Blood components were cross-matched before surgery. Given the possible need for a cesarean hysterectomy in case of uncontrolled intraoperative bleeding, informed consent was obtained.

During the surgery, the abdomen was opened by Pfannenstiel incision. Then uterine incision was given carefully in the lower segment above the fibroid after separating the uterovesical fold of the peritoneum. A term live baby was delivered by cutting through the placenta. The baby cried immediately at birth with an APGAR (Appearance, Pulse, Grimace, Activity, and Respiration) score of 9/10. The placenta and the membranes were separated easily. A central cervical fibroid measuring approximately 10 cm x 8 cm was observed that was extending posterolaterally. After placental separation, profuse bleeding from the placental bed was noticed, for which hemostatic sutures were applied, and bilateral uterine arteries were ligated. In addition to that uterine tamponade was done using two Foley catheter bulbs of volume 50 mL each. Uterotonics were given, and bleeding was controlled. The uterus was closed in two layers. A pelvic drain was put, and the abdomen was closed in layers. Intraoperatively, two packed RBCs were transfused, and postoperatively the patient was managed in the high dependency unit. The rest of the postoperative course was uneventful. The patient was discharged on the fifth postoperative day with advice for follow-up of the cervical fibroid in the postpartum period after six weeks.

## Discussion

The concurrence of uterine fibroids in pregnancy is uncommon. The reported prevalence ranges from 1.6% to 10.7% [7,8]. The cervical fibroids are the rarest type accounting for less than 1% of all fibroids reported during pregnancy [5]. Literature about cervical fibroids in pregnancy is limited to case reports and case series. They are usually asymptomatic but sometimes may significantly affect the course and outcome of pregnancy [6].

Studies have shown an increased risk of having placenta previa in cases of fibroid uterus in pregnancy [3,4,9,10]. One of the studies mentioned that uterine leiomyoma of size 5 cm or more increases the risk of placenta previa by 3.53-fold [11]. However, there is no mention of the types of fibroid typically involved in abnormal placentation. Furthermore, whether cervical fibroid has got any role in abnormal placental implantation is not known. There is only one case report of multiple cervical fibroids in pregnancy complicated by placenta previa [12].

There is no doubt that the coexistence of cervical fibroid with placenta previa is a challenge for the obstetrician as both conditions may give rise to life-threatening complications in the peripartum period. Some of the notable complications in pregnancy associated with cervical fibroid are urinary retention due to pressure effect on the bladder or urethra, degeneration of fibroid, preterm labor, obstructed labor, and postpartum hemorrhage [6]. Moreover, the concomitant presence of placenta praevia with cervical fibroid increases the risk of complication multifold. Massive hemorrhage during the peripartum period is the most fearsome life-threatening complication in this scenario. Hence, these cases should be managed in tertiary care hospitals with intensive care facilities, multidisciplinary care, and round-the-clock availability of blood products. Operative delivery is the way forward in this situation, as vaginal delivery is practically impossible, especially in cases of central placenta praevia. However, the lower segment cesarean section is challenging due to the presence of cervical fibroid on the incision line, which may cause torrential bleeding if the fibroid is accidentally incised [13]. Besides that, if placenta praevia is anterior, then the fetus can only be delivered by cutting through the placenta, which aggravates the bleeding further. The operating team should be prepared with blood products to combat the massive hemorrhage. Medical and conservative measures to control bleeding should be employed step-wise. Apart from uterotonics, procedures like balloon tamponade of the uterus, ligation or embolization of uterine artery, and internal iliac artery should be implemented to control the bleeding [14]. Cesarean hysterectomy should be considered when conservative management fails or as first-line management in cases of morbidly adherent placenta. The surgical team must prepare early for hysterectomy in anticipation preoperatively [14]. Post-operatively patient needs to be monitored in a high dependency unit as there is a possible need for massive blood transfusion and associated complications.

The decision of myomectomy during the cesarean section is fraught with danger given the risk of hemorrhagic complications and increased likelihood of hysterectomy [6]. In situations where the fibroid is associated with a placenta previa, not many obstetricians will dare to remove the fibroid. However, myomectomy during cesarean section has been reported with no major complications [15]. In usual circumstances, fibroid management must be conservative during cesarean section, particularly when associated with placenta previa. Later, myomectomy, uterine artery embolization, or hysterectomy can be contemplated depending upon the patient’s symptoms, fertility desire, and the site of the mass [16].

The current case presented to our emergency in active labor with antepartum hemorrhage. The patient had a previous ultrasound report showing a large cervical fibroid. An ultrasound done in our center revealed a central placenta praevia and a central cervical fibroid which warranted an emergency cesarean section. We anticipated massive postpartum hemorrhage, and we prepared ourselves accordingly. Intraoperatively, the uterine incision was given above the cervical fibroid in the lower uterine segment by stretching it, and then the placenta was divided to deliver the baby. As expected, there was excessive bleeding, which was managed sequentially with uterotonics, hemostatic sutures, intrauterine balloon tamponade, and uterine artery ligation. A failure in arresting the bleeding would have prompted us to consider internal artery ligation or hysterectomy.

## Conclusions

Management of large cervical fibroid with central placenta praevia presenting in antepartum hemorrhage is demanding for obstetricians. It needs careful evaluation, adequate preparedness for unexpected complications, and prompt management to save the life of the mother and fetus.

It is vital to determine the location of the fibroid in relation to the placenta and cervix before cesarean section by preoperative ultrasonography, so that incision in the lower uterine segment can be demarcated. Ideally, the incision line should not pass over the cervical fibroid to prevent massive blood loss. Myomectomy during cesarean section should be avoided for all practical purposes.

Placenta praevia is known to cause massive postpartum hemorrhage. Delivery should be conducted in obstetric setups with blood bank facilities, skilled clinicians, and critical care services. Postpartum hemorrhage can be managed efficiently by uterotonics. If pharmacotherapy fails, conservative interventions like applying hemostatic sutures, employing uterine tamponade, and performing devascularization procedures in a step-wise manner should be immediately undertaken. Interventional radiological techniques can also be employed to arrest bleeding where facilities are available. However, in case of failure of conservative methods, hysterectomy should be the way forward as a life-saving measure.
